# Breastfeeding experiences of women with perinatal mental health problems: a systematic review and thematic synthesis

**DOI:** 10.1186/s12884-024-06735-1

**Published:** 2024-09-06

**Authors:** Hayley Billings, Janet Horsman, Hora Soltani, Rachael Louise Spencer

**Affiliations:** 1 College of Health, Wellbeing and Life Sciences, Sheffield Hallam University, Collegiate Campus, Sheffield, S10 2DN UK; 2 Nursing and Midwifery College of Health, Wellbeing and Life Sciences, Sheffield Hallam University, Collegiate Campus, Sheffield, S10 2DN UK

**Keywords:** Breastfeeding, Perinatal, Mental health, Women

## Abstract

**Background:**

Despite its known benefits, breastfeeding rates among mothers with perinatal mental health conditions are staggeringly low. Systematic evidence on experiences of breastfeeding among women with perinatal mental health conditions is limited. This systematic review was designed to synthesise existing literature on breastfeeding experiences of women with a wide range of perinatal mental health conditions.

**Methods:**

A systematic search of five databases was carried out considering published qualitative research between 2003 and November 2021. Two reviewers conducted study selection, data extraction and critical appraisal of included studies independently and data were synthesised thematically.

**Results:**

Seventeen articles were included in this review. These included a variety of perinatal mental health conditions (e.g., postnatal depression, post-traumatic stress disorders, previous severe mental illnesses, eating disorders and obsessive-compulsive disorders). The emerging themes and subthemes included: (1) Vulnerabilities: Expectations versus reality; Self-perception as a mother; Isolation. (2) Positive outcomes: Bonding and closeness; Sense of achievement. (3) Challenges: Striving for control; Inconsistent advice and lack of support; Concerns over medication safety; and Perceived impact on milk quality and supply.

**Conclusions:**

Positive breastfeeding experiences of mothers with perinatal mental health conditions can mediate positive outcomes such as enhanced mother/infant bonding, increased self-esteem, and a perceived potential for healing. Alternatively, a lack of consistent support and advice from healthcare professionals, particularly around health concerns and medication safety, can lead to feelings of confusion, negatively impact breastfeeding choices, and potentially aggravate perinatal mental health symptoms. Appropriate support, adequate breastfeeding education, and clear advice, particularly around medication safety, are required to improve breastfeeding experiences for women with varied perinatal mental health conditions.

## Background

Breastfeeding is a key public health measure, conferring short- and long-term health and socio-economic benefits for women and their offspring [1–4]. Breastfeeding has been identified as crucial in meeting the United Nations Sustainable Development Goals for 2030 [5] with the World Health Organisation aiming for global rates of 50% exclusive breastfeeding until 6 months of age by 2025 [6]. Despite an increasing research base about what helps or hinders breastfeeding, there is a dramatic drop in breastfeeding prevalence within the first six weeks of birth, especially in high income countries [1, 7–9]. The reasons given for cessation of breastfeeding suggest that few mothers gave up because they planned to, citing challenges such as physical pain [10], perceived insufficient milk supply [11], and breastfeeding not fitting in with family and/ or work life [12], and although complex physiological and psychosocial factors influence breastfeeding practices, evidence also suggests that mothers who experience postnatal depression may be at a greater risk of early breastfeeding cessation [13, 14].

Perinatal mental health (PMH) conditions are mental illnesses which occur during pregnancy and up to a year following birth [15, 16] and include a range of conditions such as: depression, anxiety, obsessive compulsive disorder, post-traumatic stress disorder (PTSD), tokophobia, bipolar disorder, postpartum psychosis, eating disorders and personality disorders [17]. These conditions are associated with increased morbidity and are a leading cause of maternal death in high-income countries [17]. Globally it is estimated that between 15 and 25% of women experience mental illness during the perinatal period, either as a new condition or as a reoccurrence of a pre-existing condition [17].

Breastfeeding is known to have psychological benefits, such as improving mood and protecting against postnatal depression in mothers, enhancing socio-emotional development in the child and strengthening mother-child bonding [13, 14, 18, 19]. However, previous reviews of women’s experiences of breastfeeding whilst experiencing mental health conditions have focused primarily on postnatal depression (PND) [19]. No previous reviews have been identified which investigate the experiences and perspectives of women with a variety of perinatal mental illnesses with a view to improving breastfeeding health intervention strategies for women with such conditions.

## Methods

This systematic review was reported in accordance with the PRISMA 2020 statement [20]. The review protocol was registered with PROSPERO in 2021 (registration number CRD42021297076 Available from: https://www.crd.york.ac.uk/prospero/display_record.php?ID=CRD42021297076). There was no requirement to deviate from this protocol during the study.

### Search strategy

A literature search was undertaken for studies published from 2003 to Nov 2021. The selection of 2003 was to identify research undertaken following publication of the World Health Organisation *Global Strategy for Infant and Young Child Feeding* [21]. This advised that women exclusively breastfeed for six months and continue breastfeeding for two years and beyond for optimal health benefits to mother and infant.

The search was conducted using five electronic databases: Medline and CINAHL Complete (EBSCOhost), Maternity & Infant Care (Ovid), APA PsycInfo^®^ (ProQuest) and Web of Science Core Collection (Clarivate).

Search terms were devised according to the SPIDER (Sample, Phenomenon of Interest, Design, Evaluation, Research type) framework [22] (Table 1). Reference lists of included articles were scrutinised for possible additional studies.

**Table 1:** Search terms

SPIDER Tool	Search terms (in title or abstract)
(S) Sample: women with mental health conditions	depress* OR “mentally ill” OR distress OR bulimi* OR “dissociative disorder*” OR “post traumatic stress” OR psychosis OR anxiety OR anorexi* OR “eating disorder*” OR “mood disorder*” OR phobi* OR tocophobia OR tokophobia OR “obsessive compulsive disorder*” OR schizophrenia OR bipolar OR “mental ill health” OR “mental illness” OR “mental health” OR “adjustment disorder*” OR psychiatr*
(P) Phenomenon of Interest: Breastfeeding experiences and opinions	“breast fe*” OR “breastfe*” OR lactat* OR “Infant feed*”
(D) Design	observ* OR “case stud*” OR “focus group*” OR interview OR survey OR questionnaire*
(E) Evaluation	opinion* OR perception* OR attitude* OR perspective* OR experience* OR view*
(R) Research type	“qualitative” OR “mixed method*”

### Eligibility criteria

Eligible studies included:

i. published from 2003.
ii. peer-reviewed articles.
iii. published in English.
iv. any setting.
v. qualitative primary research data.
vi. participants were women experiencing mental health issues.
vii. described experiences, perceptions, views, and opinions in relation to breastfeeding.

### Study selection

Titles, abstracts, and potentially relevant full texts were screened independently by two authors against the eligibility criteria. Disagreement was resolved through discussion and consultation with a third author.

### Data extraction

Data extracted included study authors, title, year of publication, country of origin, source of funding, study aims, study design, recruitment strategies, participant ethnicity, PMH condition, and study results. Two authors independently extracted data.

Quality appraisal of included studies was carried out to demonstrate rigour, using a Critical Appraisal Skills Programme (CASP) appraisal tool [23], however this was not used as an indicator for inclusion in the analysis.

### Data synthesis

Thematic synthesis, a method of analysis widely used for qualitative systematic reviews, was undertaken [24]. This involved line by line coding of extracted quotations followed by development of descriptive and analytical themes. NVivo software was used to systematically code extracted data. Verbatim quotations, along with information on themes and sub-themes they were assigned to in the original study, were imported into the software. Codes and their supporting data were reviewed to identify related categories which could be grouped into broader descriptive themes. From this, overarching analytic themes were identified.

Author reflexivity was considered and addressed throughout the review with regular discussions between authors to debate and establish aspects such as definitions of mental health, use of terminology, themes, subthemes and the interplay between them.

### Patient and public involvement

Once key findings were established, the project team organised two patient and public involvement events, which included ethnic minority perinatal peer supporters and a pre/postnatal peer support group with PMH experiences. Feedback from these groups showed that the themes identified by the review captured the main priorities of the groups.

**Table 2:** Summary of CASP quality assessment

First author	Clear statement of aims?	Qualitative methodology used?	Appropriate research design?	Appropriate recruitment strategy?	Data collection	Relationship between researcher and participants considered	Ethical issues	Rigorous data analysis	Clear statement of findings	Overall quality rating
Baker	Yes	Yes	Yes	Yes	Yes	Yes	Yes	Yes	Yes	High
Beck	Yes	Yes	Yes	Yes	Yes	Yes	Yes	Yes	Yes	High
Burton	Yes	Yes	Yes	Self-selected sample	Yes	Yes	Yes	Yes	Yes	High
Coates	Yes	Yes	Yes	Yes	Yes	Yes	Yes	Yes	Yes	High
Edhborg	Yes	Yes	Yes	Yes	Yes	Can’t tell	Yes	Yes	Yes	High
Emerson	Yes	Yes	Yes	Yes	Yes	Yes	Yes	Yes	Yes	High
Fooladi	Yes	Yes	Yes	Eligibility and recruitment strategy unclear	Yes	Yes	Yes	Yes	Very little detail	Low
Gordon	Yes	Yes	No theoretical basis described for analysis of qualitative data	Secondary analysis, limited by the availability of data	Limited by data availability may not have achieved saturation	Limited	Minimal detail	Moderate detail only	Yes	Moderate
Hesse Tyson	Yes	Yes	Yes	Yes	Yes	Limited discussion on researcher bias and influence	Yes	Yes	Yes	Low
Homewood	Yes	Yes	Yes	Yes	Yes	Yes	Yes	Yes	Yes	High
Humphries	Yes	Yes	Yes	Yes	Yes	Can’t tell	Yes	Yes	Yes	High
Olson	Yes	Yes	Yes	Yes	Yes	Can’t tell	Yes	Yes	Yes	Low
Pratt	Yes	Yes	Yes	Yes	Yes	Yes	Yes	Yes	Yes	High
Scorza	Yes	Yes	Yes	Yes	Yes	Yes	Yes	Yes	Yes	High
Shakespeare	Yes	Yes	Yes	Yes	Yes	Yes	Yes	Yes	Yes	High
Stapleton	Yes	Yes	Yes	Yes	Yes	Yes	Yes	Yes	Yes	High
Stelson	Yes	Yes	Yes	Yes	Yes	Limited detail	Consent not mentioned	Yes	Yes	Moderate

## Results

**Fig. 1:**
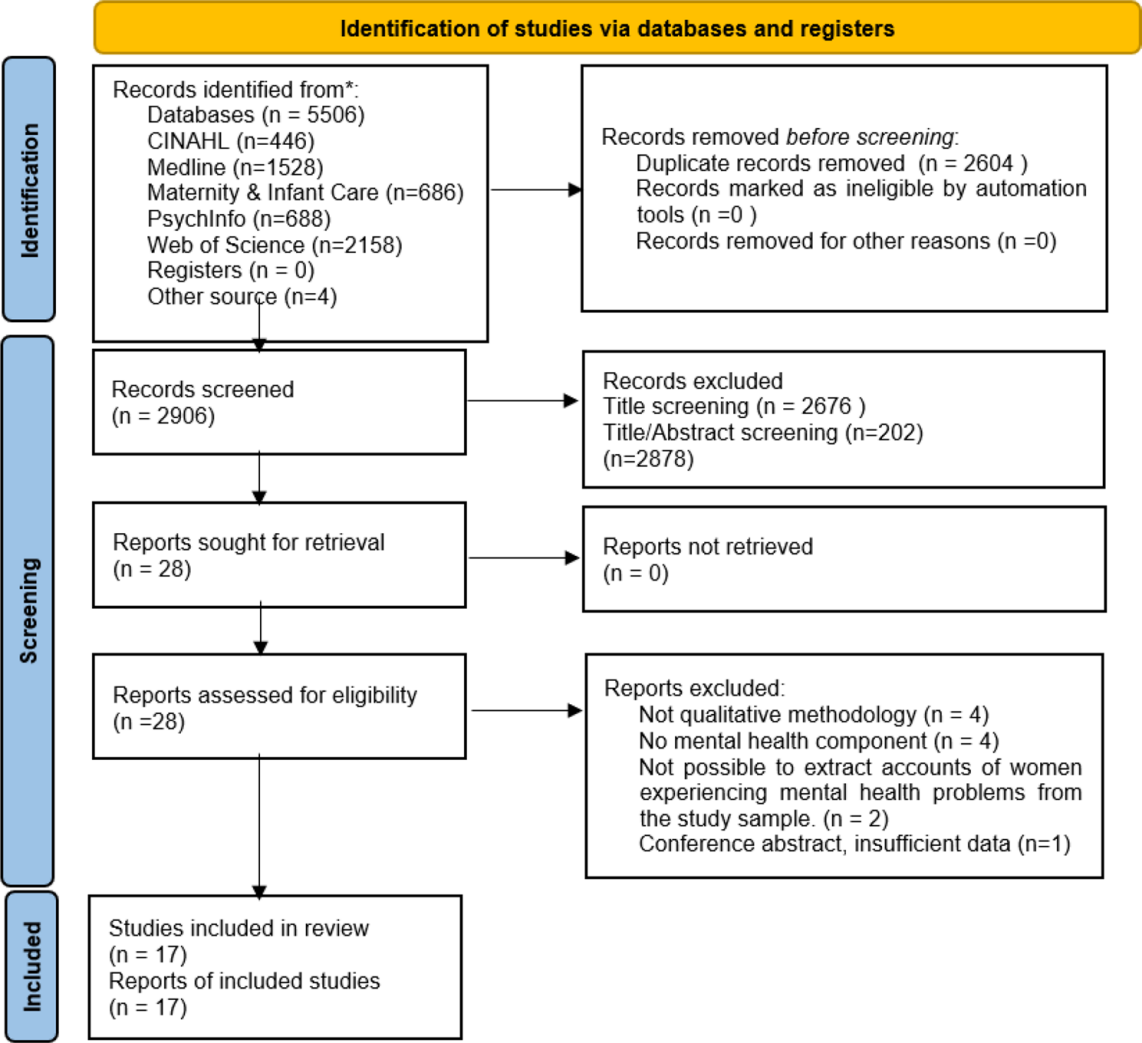
PRISMA flow diagram detailing study selection [20]. CINAHL – Cumulative Index to Nursing and Allied Health Literature. PRISMA flow diagram- Page MJ, McKenzie JE, Bossuyt PM, Boutron I, Hoffmann TC, Mulrow CD, et al. The PRISMA 2020 statement: an updated guideline for reporting systematic reviews. *BMJ*. 2021;372(71). DOI: 10.1136/bmj.n71

The study selection process is outlined on the PRISMA [20] flow diagram (Fig. 1). A total of 5510 studies were retrieved. After removing duplicates (*n* = 2604) and excluding articles which were not relevant following screening of title and abstract (*n* = 2878), full text of the remaining 28 studies were screened. Of these, 11 studies were excluded, resulting in 17 studies being included in this review.

### Characteristics of the studies

From the 17 included studies, four used thematic analysis, two in a qualitative study [25, 26] and two within a mixed methods secondary analysis of existing data [27, 28]. Six studies used phenomenological methods [29–34], two used an ethnographic approach [35, 36] and three undertook a Grounded Theory approach [37–39]. One study used a psychoanalytically informed analysis [40] and one used comparative analysis [41].

Following CASP quality appraisal, the methodological quality of included papers was ranked as either low (*n* = 3), moderate (*n* = 2) or high (*n* = 12), (Table 2).

Of the included studies, seven focused on PND, four included patients with PND and/or emotional difficulties, postnatal blues or mental distress, two focused on mood disorders, four included women previously diagnosed with severe mental illnesses, eating disorders, obsessive compulsive disorder, and/or traumatic childbirth/PTSD.

There were a total of 551 participants across the studies. Of these, 456 were married/cohabiting, 18 were single/separated, and 77 did not specify. For educational attainment, 321 participants identified as either ‘well educated’ or having studied beyond high school level. A total of 86 participants received a school education (high school or below), 14 participants had no schooling and 130 did not specify. Of the 17 studies, 15 were carried out in high-income countries and two in low-income countries (Table 3).

**Table 3:** Characteristics of included studies (*n* = 17)

First author & year	Country	Mental health condition (as defined in the paper)	Design/Methods	Number of participants	Ethnicity of participants	Education status	Marital status
Baker (2021)	UK	Severe mental illness	Mixed methods secondary analysis of existing data	218 (25 in qualitative analysis)	168 White 50 non-Caucasian	38 School 153 Higher Education 27 Degree	201 married/ in relationship during pregnancy
Beck (2008)	USA, New Zealand, Australia, UK, & Canada	Traumatic childbirth 37% PTSD diagnosis	Phenomenology	52	Not stated	2 not stated 4 High School 38 College 8 Graduate school	46 married 5 cohabiting 1 separated
Burton (2021)	UK	Obsessive Compulsive Disorder	Phenomenology	5	White British	4 Bachelor’s degree 1 Foundation diploma	3 married 1 cohabiting 1 single
Coates (2014)	UK	Emotional difficulties	Phenomenology	17	16 White 1 Chinese	4 Professional 9 Degree 6 GCSE/A level	Not stated
Edhborg (2005)	Sweden	PND	Grounded theory	22	Not stated	13 University 9 secondary school	13 married 9 cohabiting
Emerson (2017)	Democratic Republic of the Congo	Mental distress	Grounded theory	35	32 African 3 not stated	14 none 11 primary 9 secondary	33 married 2 not stated
Fooladi (2006)	USA	Postnatal blues	Ethnography	9	White	Not stated	Not stated
Gordon (2021)	USA	Major Depressive Disorder or Bipolar	Mixed methods secondary analysis of existing data	48 (9 in qualitative analysis)	34 White 5 Latina 5 Multi-racial 3 Black 1 Asian	9 High school or below 25 College/trade school 14 graduate /degree/ professional	39 married/in a relationship
Hesse Tyson (2020)	Ireland	PND	Psychoanalytically informed analysis	6	Not stated	Not stated	5 cohabiting 1 single
Homewood (2009)	UK	PND	Grounded theory	9	White British	Not stated	7 married 1 cohabiting 1 single
Humphries (2012)	Canada	Mood disorder (under psychiatrist)	Phenomenology	6	White	Not stated	Not stated
Olson (2014)	Canada/ USA	PND	Phenomenology	5	Not stated	‘well educated’	5 married/ cohabiting
Pratt (2020)	USA	PND	Phenomenology	10	9 White, 1 African American	2 college 2 associate degree 6 Degree	6 married 2 cohabiting 1 single
Scorza (2015)	Ghana	PND	Comparative analysis	42	Not stated	Not stated	All married/ cohabiting
Shakespeare(2004)	UK	PND/emotional difficulties	Qualitative	39	37 White 2 other	Not stated	35 cohabiting 4 single
Stapleton (2008)	UK	Eating disorders	Ethnography	16	White	Not stated	Not stated
Stelson (2021)	USA	PND	Qualitative	12	8 Black/African American, 1 Latina, 3 more than one race	9 degree, 6 college, 3 high school or below	3 married 9 single

GCSE – General Certificate of Secondary Education

PND – Postnatal Depression

PTSD – Post traumatic stress disorder

### Themes

**Fig. 2:**
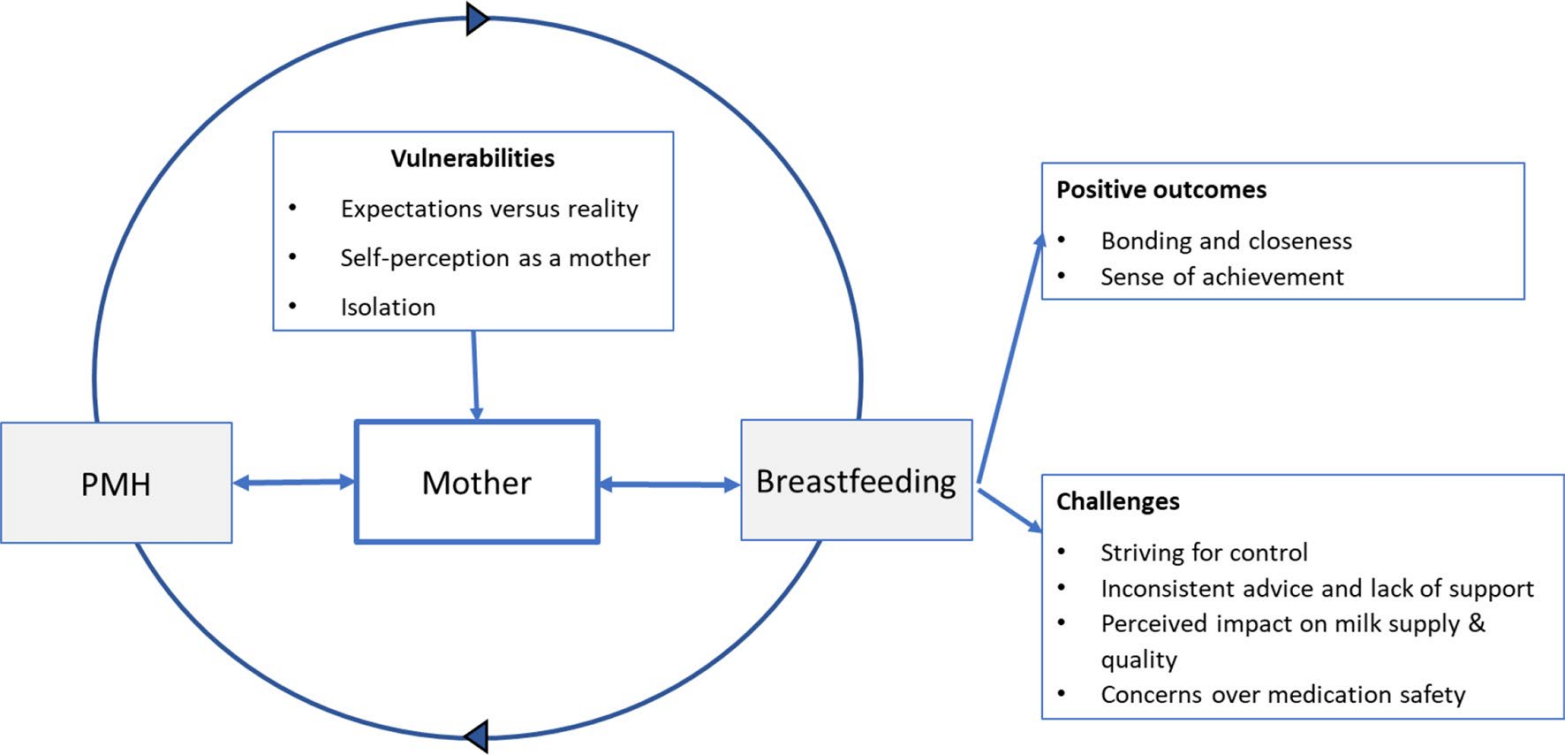
Illustration of the interplay of themes and subthemes of the breastfeeding experiences of women with perinatal mental health problems PMH – Perinatal mental health

Through in-depth analysis of the data, three overarching themes: Vulnerabilities, Positive outcomes, and Challenges, emerged. These themes and associated sub themes are shown in Table 4. The interplay among these major domains within the context of themes and subthemes are summarised in Fig. 2.

**Table 4:** Representation of themes and subthemes across the included studies

First author	Bonding & closeness	Sense of achievement	Expectation v Reality	Self-perception as a mother	Isolation	Striving for control	Inconsistent advice & lack of support	Concerns over medication safety	Perceived impact on milk quality & supply
Baker							Y	Y	
Beck	Y	Y		Y		Y			Y
Burton				Y	Y	Y	Y		
Coates			Y	Y	Y	Y	Y		
Edhborg			Y	Y	Y		Y		
Emerson							Y		Y
Fooladi					Y	Y			
Gordon			Y	Y		Y	Y		
Hesse Tyson	Y	Y	Y	Y	Y		Y		
Homewood	Y			Y					
Humphries			Y		Y		Y	Y	
Olson	Y	Y	Y	Y	Y	Y	Y		
Pratt	Y	Y		Y	Y		Y		
Scorza							Y		Y
Shakespeare		Y	Y	Y		Y	Y		
Stapleton	Y	Y		Y		Y	Y		Y
Stelson	Y	Y	Y			Y		Y	

### Theme: vulnerabilities

#### Expectations versus reality

For some new mothers the reality of breastfeeding did not meet their expectations of being easy and ‘natural’, leaving them feeling unprepared and disillusioned when they experienced difficulties.

“You think you’re a completely useless mother and, you know, you should be able to know how to do this instinctively [breastfeeding] and in fact it’s probably the hardest thing I’ve ever done.” (25, p255).

Limited availability of antenatal breastfeeding advice led to mothers being unaware of the potential complexities of breastfeeding during the early days and weeks.

“Everyone make it seem like it’s natural because your body produces [milk]. It’s just something that should frequently come to you as soon as you have the baby., but it’s not like that. You had to hold the baby a certain way, you got to adjust your thing a certain way, you got to put the nipple in far enough for the baby to get it. There’s a lot to it. It’s really complicated.” (26, p5).

### Self-perception as a mother

To be perceived as a ‘good mother’, by themselves and others, some women felt they must breastfeed at all costs. This perceived association of breastfeeding as the representation of ‘good mothering’, appeared to result in self-imposed pressure.

“I was so desperate to breastfeed him and I felt as if it was my, I felt as if I had some moral obligation as a mother and if I didn’t breast feed him I was badly letting him down.” (25, p255).

If these women were then unable to breastfeed, or if they faced significant breastfeeding difficulties, this sometimes led to feelings of guilt or inadequacy.

“There’s so much pressure on you to breastfeed. so you’re told that breast is best and you should do it and so when you don’t you think you are a failure and it’s what you should be doing.” (39, p322).

The opinions of family, friends and health professionals also played a significant part in the woman’s perception of her status as a ‘good mother’.

“The approval thing was a big factor. Everyone was telling me how well I’d done to keep breastfeeding. All that approval made me feel really good about myself, and that I was being a good mother to (baby). I wasn’t thinking negative thoughts about myself, I was feeling very positive really.” (35, p114).

However, for some mothers, this resulted in added pressure, causing them to hide their feelings and maintain an outward display of happiness.

“I didn’t want to talk with anybody about it, I always had to pretend that I was doing just great … I thought that wasn’t normal, that I was a bad mom who felt that way.” (37, p264).

Finding the right support could be very beneficial but some women had negative experiences of clinics or groups, undermining their self-belief.

“Daggers are drawn and everybody’s acting as if they can rule the world and the trouble is, when you’re depressed you just see that image and you think, I’m never going to be as good as this.” (39, p323).

### Isolation

Feelings of isolation felt by breastfeeding women were exacerbated by mental health issues, with Homewood et al. (39, p325) suggesting that breastfeeding could contribute to depression by increasing the sense of being trapped by the infant’s dependency.

“abandoned and alone … .scared all the time that something would happen to the baby…” (37, p264).

The sense of isolation was increased by the fact that seeking help could be difficult for women who were distressed because they were reluctant to reveal their negative feelings.

“I was feeling like really sad and just really isolated and really stuck!. . I just thought. . “How am I going to take care of this baby? And I am feeling so crappy!” I found it to be really hard just to reach out and admit that I was feeling the way that I was. I don’t know why I was so worried about being stigmatized, but I was. I just didn’t want that label of being a person with postpartum depression.” (32, p12) “.

### Theme: positive outcomes

#### Bonding and closeness

Whilst struggling with mental health issues, the experience of breastfeeding successfully could increase mothers’ positive feelings toward the baby, allowing them to enjoy time spent together and enhance their confidence.

“I used to feed her and it was the time I got a little lump in my throat and thought, oh, perhaps she’s not that bad, and I thought, this is perhaps how people feel a bit more of the time than I feel it.” (39, p323).

Some women reported that the physical aspect of breastfeeding allowed a connection that could compensate, to a degree, for the mental withdrawal caused by the depressive symptoms.

“I think [breastfeeding] helps because even if I feel like some days I’m not very connected emotionally, I know that at least I’m providing the baby with physical touch and bonding and all that. Even if I’m not mentally 100% there. So, I think it makes me feel better about myself as a mom.” (33, p641).

One mother noted that breastfeeding could reduce feelings of stress.

‘‘When I’m nursing her, I’m able to just hold her. And that just alleviates any worries, any stress that I’ve had through the day, just knowing that she needs me, that she’s finding comfort in me, that I’m able to comfort her. She’s comforting me at the same time.” (26, p5).

### Sense of achievement

Achieving success with breastfeeding was a factor in mitigating some of the guilt that women with eating disorders might feel about the possible effects of their eating disorder on the baby, positively affecting their self-esteem.

“It wasn’t my instinct to want to breastfeed him but in the end I did. In some ways it made up for all the damage I thought I’d done to him because of my eating disorder.” (35, p113).

Some women who had experienced a traumatic birth perceived breastfeeding as having the potential to heal and reinforced their self-perception as a good mother.

“I would cover her up to feed her and hide her little head in the clothing. Not because of dignity, but because I did not want anyone else to see the magic and healing that was happening between us. Being able to breastfeed my daughter, despite all the odds, is my proudest achievement in life. I wear it in my soul as a badge of honor.” (29, p233).

Women described how breastfeeding was within their sphere of control whereas other aspects of motherhood were not.

“[Breastfeeding] was the one thing that I could control. . I think that it made me feel better because it was the one thing that I was successful at, as a mom, because my birth went so shitty, and everything just kind of spiraled down and my mood and everything. . .I lean on [breastfeeding] a lot. It is my thing with her that no one can take away. . .I don’t like other people doing it. I don’t even like the suggestion of other people doing it.” (32, p12).

### Theme: challenges

#### Striving for control

Some women with eating disorders perceived stopping breastfeeding as the only way to allow them to resume control over their body and their eating.

“I wanted my body back and I knew I wouldn’t get it back until I’d stopped breastfeeding. I knew the minute that stopped feeding him I could control my food again and that’s what I wanted. When I was feeding I needed to eat properly because he needs the nutrients.” (35, p114).

For women with obsessive compulsive disorder [30], some responded to contamination fears by breastfeeding, sometimes for much longer than planned.

“I forced myself to breastfeed for the whole of the first year because I was convinced that formula would be contaminating his body.” (30, p317).

Other women with eating disorders chose not to breastfeed in order to allow themselves to return to purging and undertaking strenuous exercise in order to lose their pregnancy weight rapidly [35].

Some still struggled between eating a ‘good’ diet to produce ‘healthy’ milk and the desire to return to their usual strategies such as restricted eating or purging.

“I didn’t need to make myself sick so often [when breastfeeding] but that wasn’t because I didn’t want to! [Laughs] I had to fight with myself all the time to control the urge. I thought breastfeeding would take that urge away but it didn’t. It eased a bit but I was still vomiting all the time I was breastfeeding.” (35, p112).

### Inconsistent advice and lack of support

Women’s difficulties and lack of confidence with breastfeeding were increased by inconsistent advice from both professionals and family [25]. Mothers frequently made reference to seeking advice from healthcare professionals during the early weeks of breastfeeding but felt they were often left unsupported.

“I was alone and . the nurse often didn’t answer the buzzer, my buzzer when I was trying to breast feed and things. Again I felt so kind of, incredibly sensitive about everything, and anxious about everything, and they just weren’t there, were never there for me.” (25, p256).

Mothers described feeling pressurised by healthcare professionals to continue breastfeeding [35] and, without adequate support, women would often turn to friends or relatives for infant feeding advice [25].

### Concerns over medication safety

Concerns regarding medication safety and breastfeeding [26, 27, 34] led some women to discount breastfeeding as an option for them.

“….I could try and breastfeed, but yeah, I decided that wasn’t—a good idea. Because it’s too hard and I wouldn’t be able to go back on my medication—right away after the baby was born. You have to wait two months, or something like that. So I thought that was dangerous— for both of us.” (34, p383).

Whilst others discontinued breastfeeding due to health concerns for the baby.

“And I had to get my wisdom teeth pulled out, so I decided to stop because they put you on antibiotics and stuff like that. So I just stopped.”(26, p5).

Some women with severe mental illness felt that due to the complexities of their mental health, breastfeeding was not considered relevant and was “de-prioritized” for other aspects of acute care [27]. Despite many mothers expressing strong preferences to continue breastfeeding, the mothers often felt that their preferences were ignored.

“Medication was an issue as I was initially given medication that specified it should not be taken while breastfeeding, when I had made my wish to breastfeed very clear.” (27, p7).

Some women felt that they needed to prompt staff to consider whether the medication they were prescribed would allow breastfeeding, or, alternatively, be given the choice to cease breastfeeding to allow them to have the most suitable medication to treat their mental health condition.

“I wish they had told me to stop breastfeeding rather than give me diluted medication.” (27, p6).

Others described being given contradictory information from health professionals about breastfeeding whilst taking psychotropic medication:

“Early in pregnancy, the mental health midwife said not to take fluoxetine if breastfeeding and to change to sertraline or citalopram. Next time I saw her later on and she said I could stay on fluoxetine if I was happy on it.” (27, p6).

Such conflicting advice made mothers confused and distressed. A resultant lack of confidence in healthcare professionals “*prompted some women to conduct their own research or to disregard medical advice*” (27, p6).

#### Perceived impact on milk quality and supply

There was a perception that women with PMH conditions would be unable to produce a sufficient quality and/or volume of breastmilk to sustain their baby nutritionally. This concern could potentially generate feelings of depression for women [26].

Some mothers perceived that their own poor nutrition could potentially cause problems with breastfeeding. This concern was often associated with eating disorders [26], food unavailability or lack of appetite due to mental ill health [38, 41]. For women with eating disorders there was a belief that frequent cycles of binging and purging were not compatible with producing sufficient good quality breast milk. This caused some women to discount breastfeeding, and some received pressure from partners to bottle feed in the belief that the child would not receive the necessary nutrition.

“He (husband) didn’t want me to breastfeed because he thought I wasn’t eating enough to feed her (baby) properly. [.] He was on and on about me giving her the bottle. He even dragged my sister in to try and get her to talk me round.” (35, p111).

Some women with eating disorders did wish to breastfeed and commented on needing to change their eating patterns to achieve this.

“I had to eat properly when I was breastfeeding because I had a baby to think about. The baby needs nutrition. I thought whatever I eat the baby is going to get it. So I had to eat properly. Like when I was pregnant I made myself eat properly.” (35, p112).

## Discussion

Depression and anxiety are a common problem in the perinatal period, and pregnancy and childbirth can put women at risk of relapse or exacerbation of pre-existing mental illness [17]. Although postpartum anxiety is more prevalent than postpartum depression [42] we did not find any studies of women’s experiences of anxiety and breastfeeding. In this review there were examples of specific mental illnesses being associated with specific issues in relation to breastfeeding along with the difficulties faced by many women. Data from the included studies replicated what is already known regarding the relationship between perinatal depression and breastfeeding, that this relationship is bidirectional, with evidence of depressive symptoms contributing to worse breastfeeding outcomes and breastfeeding challenges sometimes serving as a trigger for postnatal depressive symptoms [43].

In this study it was found that, for mothers who were struggling with their mental health, the sense of achievement obtained by successful breastfeeding could boost their self-esteem and bolster the perception of themselves as a good mother [29, 32, 40]. These mothers found that breastfeeding could increase their mother/child bond and reinforce their confidence as a mother and felt that the closeness experienced during breastfeeding could reduce feelings of stress and compensate their baby for times when they were feeling withdrawn [26, 33, 35, 39].

However, the perception that ‘good mothering’ is defined by successful breastfeeding can also result in overwhelming pressure for mothers, who may feel obliged to breastfeed despite experiencing challenges [44–46]. This pressure can then be further compounded by the attitudes and behaviours of healthcare professionals, family members and society in general [46]. A large proportion of the women in the included studies had a strong intention to breastfeed [25, 28, 31, 33, 37] and were often motivated to continue, despite difficulties, because of the pressure they placed on themselves to fulfil the role of the ‘good mother’. If they then had difficulties or ceased breastfeeding they often experienced feelings of guilt, inadequacy, and failure [25, 27, 40].

There is a wealth of literature describing the guilt and despair experienced when women’s expectations for breastfeeding to occur naturally, the desire to be a good mother, and ‘breast is best’, clash with the demands and labour-intensive workload that breastfeeding often entails [43, 44, 47, 48]. A lack of antenatal education regarding potential breastfeeding challenges appears to be evident, with much of this being dedicated to the benefits of breastfeeding to both mother and baby, and although this information is important, it can provide a skewed ideal of the breastfeeding process [47]. Findings from studies by Hoddinott et al. [47] and Redshaw and Henderson [48] suggested realistic antenatal education is key to preparing women for common difficulties and suggest providing a realistic view rather than rosy pictures or patronising breastfeeding workshops with knitted breasts and dolls [47]. This lack of preparation for the challenges that frequently arise during the early days of breastfeeding can result in mothers feeling inadequate and unable to cope [45, 46], potentially resulting in early discontinuation of breastfeeding and/or a decline in mental wellbeing.

The perception that mental health conditions can lead to insufficient or poor-quality breast milk is a common perception amongst breastfeeding women. A systematic review of breastfeeding problems by Karaçam and Sağlık, [49], found that 12 out of 34 studies referred problems such as “inadequate breastmilk/lack of breastmilk/ concern for inadequate breastmilk/thought that the baby was not satiated adequately/inadequate weight gain.” The theme was again identified by this study, particularly amongst those with eating disorders [29, 35] and women from the two African based studies [38, 41]. Women’s perceptions were primarily that poor mental health leads to inadequate nutritional intake (due to lack of appetite/disordered eating) and therefore impacts breastmilk volume and quality. This added burden of believing that their breastmilk may not adequately sustain their child could potentially further impact their mental health as a perceived failure [29].

For some women a sense of isolation in their role as carer, and specifically regarding breastfeeding was expressed in the included studies [25, 32, 34]. The sense of isolation can be magnified both by the symptoms of mental health issues and the reluctance of the mothers to reveal their condition, either pre-existing or newly emerging, to their loved ones and health professionals, worrying about what they may think [30, 32, 37]. This is reflected in previous research, which found stigma associated with mental ill health, compounded by a pervasive social stigma attached to being seen to ‘fail’ as a mother, leads to under-reporting of perinatal mental health issues [50]. A study of Australian women undergoing routine psychosocial assessment also found that 11.1% reported they were not always honest in the assessment and lack of trust in the midwife was the most frequent reason for non-disclosure [51]. Failure to reveal previous mental health issues may lead to inappropriate or sub-optimal advice [35].

The findings also identified that a lack of trust in the support and advice given by health professionals was also a contributing factor when considering medication safety and was stated as a reason to cease or not commence breastfeeding [27, 34]. These inconsistencies sometimes prompted women to undertake their own research to gain answers [27], which could potentially lead to serious health consequences. The concerns held by women regarding medication safety and breastfeeding were highlighted in a Swedish study [52] which found that 57.7% of pregnant participants classed medication use during breastfeeding as harmful/probably harmful.

This lack of consistent advice regarding medication safety is largely due to a lack of high-quality evidence [53]. However, for those requiring medication during the postnatal period, clearer guidance is needed from healthcare professionals on the suitability of each type of medication when breastfeeding, and whether alternative medications can be considered so that breastfeeding can be undertaken safely without additional worry. Some women in the study felt they did not have the opportunity to make an informed choice regarding their medication and that desire to breastfeed was deprioritised over their mental health [27]. However, findings from this and previous studies have shown that when breastfeeding is successful it can improve mood and help protect against postnatal depression [32, 33, 54, 55], as well as strengthen mother-child bonding [26, 33]. It may therefore be the case that, in conjunction with suitable medication, breastfeeding may further help to boost mood and improve the overall wellbeing of the mother by providing a sense of achievement and control.

Negative attitudes towards diagnosis and treatment of perinatal mental health conditions result in women avoiding help seeking and reinforces feelings of stigma and guilt. Organisational-level factors such as inadequate resources, fragmentation of services and poor interdisciplinary communication compound these individual-level issues [50, 56]. Structural factors (especially poor policy implementation) and sociocultural factors (for example language barriers) also cause significant barriers to accessing services for this group of women [50, 56].

A strength of this review is the inclusion of literature regarding various mental health conditions (not purely depression) which had not been previously synthesised. This review highlights that each mental health condition may impact differently on breastfeeding experiences and merits separate investigation to inform policy and practice. The findings from this synthesis were based on a systematic literature search of five electronic databases. Inductive and in-depth analysis, using an iterative approach, allowed for immersion in the data, which strengthened the review findings.

Limitations were similar to those identified by previous studies relating to maternal mental health needs [57]. Participants were predominantly white and well educated, and studies were primarily undertaken in high income countries. This means that the findings may not be applicable to all women particularly those from low-income countries who may have different experiences and needs. None of the included studies incorporated the views and experiences of women from low socioeconomic status specifically, who are more likely to experience PMH conditions [47]. A comparison of the breastfeeding experiences of women with PMH conditions between different countries was beyond the scope of this review, however it must be acknowledged that differences are expected due to variations in culture, health systems, resource, and infant feeding attitudes.

The methods of diagnosing mental health conditions differed between studies. Some participants had a clinical diagnosis, whilst some were included based upon tools such as the Edinburgh Postnatal Depression Scale, or a self-diagnosis of distress/depression. This allowed us to increase the scope of studies included but may mean that some studies included women who may have not met the criteria for a clinal diagnosis of depression.

To ensure completeness prior to publication, the original search was again undertaken to capture any studies published between November 2021 and February 2024. The search identified two further papers which met the inclusion/exclusion criteria. Both papers supported the original themes found in the study and therefore further validated the findings. Scarborough et al. [58] reported a perceived pressure to breastfeed, mixed impact on the mental health of the mother and the mother infant bond, and challenges receiving adequate information and support. Frayne et al. [59] highlighted the importance of good communication, consistency of advice, and shared decision making for women taking psychotropic medication, and the challenges faced if these aspects were not achieved.

## Conclusions

There is a complex dynamic relationship amongst breastfeeding intention, practice, and experiences for mothers with PMH conditions. The intensity and magnitude of positive outcomes that women describe, and the challenges experienced, are exacerbated in mothers with PMH conditions. The challenging experiences are particularly influenced by a lack of support, shame, fear of stigmatisation and additional health concerns, such as worries over medication safety.

The synthesis identified inconsistent advice from healthcare professionals, particularly in relation to medication. Further training and improved communication pathways between specialities may help enhance perinatal maternity care provision. An in depth understanding of the women’s views/needs in relation to their specific PMH condition could help enhance their experiences of infant feeding. This will help women to make informed choices about feeding, increasing their sense of control and improving self-efficacy, which could have a positive impact on their emotional and physical wellbeing, their ability to bond with their baby and their transition to motherhood.

Gaps identified through this systematic review include the need for further investigation on breastfeeding and PMH in women from minority groups, as well as a need for robust evidence and advice on medication use during breastfeeding for women experiencing perinatal mental ill health.

## Data Availability

All data generated or analysed during this study are included in this published article [and its supplementary information files].

## References

[1] Victora CG, Bahl R, Barros AJ, Franca GV, Horton S, Krasevec J, Murch S, Sankar MJ, Walker N, Rollins NC. Lancet Breastfeeding Series Group. Breastfeeding in the 21st century: Epidemiology, mechanisms and lifelong effect. Lancet. 2016;387(10017):475–90. 10.1016/S0140-6736(15)01024-7. 26869575 10.1016/S0140-6736(15)01024-7

[2] Horta BL, Loret de Mola C, Victora CG. Breastfeeding and intelligence: a systematic review and meta-analysis. Acta Paediatr. 2015a;104:14–9. 10.1111/apa.13139. 26211556 10.1111/apa.13139

[3] Horta BL, Loret de Mola C, Victora CG. Long-term consequences of breastfeeding on cholesterol, obesity, systolic blood pressure and type 2 diabetes: a systematic review and meta analysis. Acta Paediatr. 2015b;104:30–7. 10.1111/apa.13133. 26192560 10.1111/apa.13133

[4] Rollins NC, Bhandari N, Hajeebhoy N, Horton S, Lutter CK, Martines JC, Piwoz EG, Richter LM, Victora CG. Lancet Breastfeeding Series. Why invest, and what will it take to improve breastfeeding practices? Lancet. 2016;387(10017):491–504. 10.1016/S0140-6736(15)01044-2. 26869576 10.1016/S0140-6736(15)01044-2

[5] United Nations. *Transforming our world: the 2030 agenda for sustainable development* https://sdgs.un.org/2030agenda [Accessed 12th May 2021].

[6] World Health Organisation. *Breastfeeding*. https://www.who.int/health-topics/breastfeeding#tab=tab_3 [Accessed 12 May 2021].

[7] McFadden A, Gavine A, Renfrew MJ, Wade A, Buchanan P, Taylor JL, Veitch E, Rennie AM, Crowther SA, Neiman S, MacGillivray S. Support for healthy breastfeeding mothers with healthy term babies. Cochrane Database Syst Reviews. 2017;2017(2):CD001141–001141. 10.1002/14651858.CD001141.pub5. 10.1002/14651858.CD001141.pub5 PMC6464485 28244064

[8] Skouteris H, Bailey C, Nagle C, Hauck Y, Bruce L, Morris H. Interventions designed to promote exclusive breastfeeding in high-income countries: a systematic review update. Breastfeed Med. 2017;12(10):604–14. 10.1089/bfm.2017.0065. 28885859 10.1089/bfm.2017.0065

[9] Public Health England. Breastfeeding prevalence at 6 to 8 weeks after birth (experimental statistics). London: TSO; 2021.

[10] Brown CR, Dodds L, Legge A, Bryanton J, Semenic S. Factors influencing the reasons why mothers stop breastfeeding. Can J Public Health. 2014;105(3):179–85. 10.17269/cjph.105.4244 PMC6972160 25165836

[11] Galipeau R, Baillot A, Trottier A, Lemire L. ‘Effectiveness of interventions on breastfeeding self-efficacy and perceived insufficient milk supply: a systematic review and meta-analysis’. Matern Child Nutr 2018;14 (3). 10.1111/mcn.12607 PMC6866142 29655287

[12] Dagher RK, McGovern PM, Schold JD, Randall XJ. ‘Determinants of breastfeeding initiation and cessation among employed mothers: a prospective cohort study’. BMC Pregnancy Childbirth. 2016;16 (194). 10.1186/s12884-016-0965-1 PMC4966748 27472915

[13] Dennis C, McQueen K. Does maternal postpartum depressive symptomatology influence infant feeding outcomes. Acta Paediatr. 2007;96(4):590–4. 10.1111/j.1651-2227.2007.00184.x. 17391475 10.1111/j.1651-2227.2007.00184.x

[14] Krol KM, Grossmann T. Psychological effects of breastfeeding on children and mothers. Bundesgesundheitsblatt. Gesundheitsforschung Gesundheitsschutz. 2018;61(8):977–85. 10.1007/s00103-018-2769-0. 10.1007/s00103-018-2769-0 PMC6096620 29934681

[15] Daehn D, Rudolf., Pawils S, Renneberg B. Perinatal mental health literacy: knowledge, attitudes, and help-seeking among perinatal women and the public – a systematic review. BMC Pregnancy Childbirth. 2022;22(1):1–574. 10.1186/s12884-022-04865-y. 35854232 10.1186/s12884-022-04865-y PMC9295513

[16] O’Hara MW, Wisner KL. Perinatal mental illness: definition, description and aetiology. Best Practice & Research. Clin Obstet Gynecol. 2014;28(1):3–12. 10.1016/j.bpobgyn.2013.09.002. 10.1016/j.bpobgyn.2013.09.002 PMC7077785 24140480

[17] Howard LM, Khalifeh H. Perinatal mental health: a review of progress and challenges. World Psychiatry. 2020;19(3):313–27. 10.1002/wps.20769. 32931106 10.1002/wps.20769 PMC7491613

[18] Tucker Z, O’Malley C. Mental Health benefits of Breastfeeding: A literature review. Curēus. 2022;14(9):e29199–29199. 10.7759/cureus.29199. 36258949 10.7759/cureus.29199 PMC9572809

[19] Tanganhito DDS, Bick D, Chang Y. Breastfeeding experiences and perspectives among women with postnatal depression: a qualitative evidence synthesis. Women Birth. 2020;33(3):231–9. 10.1016/j.wombi.2019.05.012. 31196830 10.1016/j.wombi.2019.05.012

[20] Page MJ, McKenzie JE, Bossuyt PM, Boutron I, Hoffmann TC, Mulrow CD, et al. The PRISMA 2020 statement: an updated guideline for reporting systematic reviews. BMJ. 2021;372(71). 10.1136/bmj.n71. 10.1136/bmj.n71 PMC8005924 33782057

[21] World Health Organisation. *Global strategy for infant and young child feeding*.https://www.apps.who.int/iris/bitstream/handle/10665/42590/9241562218.pdf [Accessed 10th May 2021].

[22] Cooke A, Smith D, Booth A, Beyond PICO. The SPIDER tool for qualitative evidence synthesis. Qual Health Res. 2012;22(10):1435–43. 10.1177/1049732312452938. 22829486 10.1177/1049732312452938

[23] Critical Appraisal Skills Programme. *CASP Qualitative Studies Checklist*. https://casp-uk.net/casp-tools-checklists/. [Accessed 4th June 2021].

[24] Thomas J, Harden A. Methods for the thematic synthesis of qualitative research in systematic reviews. BMC Med Res Methodol. 2008;8:45. 10.1186/1471-2288-8-45. 18616818 10.1186/1471-2288-8-45 PMC2478656

[25] Shakespeare J, Blake F, Garcia J. Breast-feeding difficulties experienced by women taking part in a qualitative interview study of postnatal depression. Midwifery. 2004;20(3):251–60. 10.1016/j.midw.2003.12.011. 15337281 10.1016/j.midw.2003.12.011

[26] Stelson EA, Kulkacek L, Frasso R, Hall M, Guevara JP. Perspectives on breastfeeding from mothers with Postpartum Depression symptoms: a qualitative Assessment of antecedents, barriers, facilitators, and intervention suggestions. Breastfeed Med. 2021;16(10):790–8. 10.1089/bfm.2020.0251. 34010030 10.1089/bfm.2020.0251 PMC8817733

[27] Baker N, Potts L, Jennings S, Trevillion K, Howard LM. Factors affecting infant feeding practices among women with severe Mental illness. Front Global Women’s Health. 2021;2:624485. 10.3389/fgwh.2021.624485. 10.3389/fgwh.2021.624485 PMC8593974 34816188

[28] Gordon LK, Mason KA, Mepham E, Sharkey EM. A mixed methods study of perinatal sleep and breastfeeding outcomes in women at risk for postpartum depression. Sleep Health. 2021;7(3):353–61. 10.1016/j.sleh.2021.01.004. 33640360 10.1016/j.sleh.2021.01.004 PMC9665349

[29] Beck CT, Watson S. Impact of birth trauma on Breast-feeding: a tale of two pathways. Nurs Res. 2008;57(4):228–36. 10.1097/01.NNR.0000313494.87282.90. 18641491 10.1097/01.NNR.0000313494.87282.90

[30] Burton HAL. How women with established obsessive compulsive disorder experience pregnancy and postpartum: an interpretative phenomenological analysis. J Reproductive Infant Psychol. 2021;39(3):313–25. 10.1080/02646838.2020.1718628. 10.1080/02646838.2020.1718628 32000519

[31] Coates R, Ayers S, de Visser R. Women’s experiences of postnatal distress: a qualitative study. BMC Pregnancy Childbirth. 2014;14:359. 10.1186/1471-2393-14-359. 25315742 10.1186/1471-2393-14-359 PMC4288655

[32] Olson T, Holstlander L, Bowen A. Mother’s milk, mother’s tears breastfeeding with postpartum depression. Clin Lactation. 2014;5(1):9–15. 10.1891/2158-0782.5.1.9. 10.1891/2158-0782.5.1.9

[33] Pratt BA, Longo J, Gordon SC, Jones NA. Perceptions of breastfeeding for women with Perinatal Depression: a descriptive phenomenological study. Issues Ment Health Nurs. 2020;41(7):637–44. 10.1080/01612840.2019.1691690. 32243211 10.1080/01612840.2019.1691690

[34] Humphries JM, McDonald C. Unveiling new dimensions: a hermeneutic exploration of perinatal mood disorder and infant feeding. Issues Ment Health Nurs. 2012;33(6):377–86. 10.3109/01612840.2012.656824. 22646202 10.3109/01612840.2012.656824

[35] Stapleton H, Fielder A, Kirkham M. Breast or bottle? Eating disordered childbearing women and infant-feeding decisions. Maternal Child Nutr. 2008;4(2):106–20. 10.1111/j.1740-8709.2007.00121.x. 10.1111/j.1740-8709.2007.00121.x PMC6860811 18336644

[36] Fooladi MM. Therapeutic tears and postpartum blues. Holist Nurs Pract. 2006;20(4):204–11. 10.1097/00004650-200607000-00009. 16825923 10.1097/00004650-200607000-00009

[37] Edhborg M, Friberg M, Lundh W, Widström AM. Struggling with life: narratives from women with signs of postpartum depression. Scand J Public Health. 2005;33(4):261–7. 10.1080/14034940510005725. 16087488 10.1080/14034940510005725

[38] Emerson JA, Tol W, Caulfield LE, Doocy S. Maternal psychological distress and perceived impact on child feeding practices in South Kivu, DR Congo. FoodNutr Bull. 2017;38(3):319–37. 10.1177/0379572117714385. 10.1177/0379572117714385 28627261

[39] Homewood E, Tweed A, Cree M, Crossley J. Becoming occluded: the transition to motherhood of women with postnatal depression. Qualitative Res Psychol. 2009;6(4):313–29. 10.1080/14780880802473860. 10.1080/14780880802473860

[40] Hesse Tyson C, O’Connor J, Sheehan JD. No space for mother’s mind: a psychoanalytically oriented qualitative study of the experiences of women with a diagnosis of postnatal depression. Int J Appl Psychoanal Stud. 2020;18(4):393–407. 10.1002/aps.1687. 10.1002/aps.1687

[41] Scorza P, Owusu-Agyei S, Asampong E, Wainberg ML. The expression of perinatal depression in rural Ghana. Int J Cult Mental Health. 2015;8(4):370–81. 10.1080/17542863.2015.1037849. 10.1080/17542863.2015.1037849 PMC4629504 26539247

[42] Zappas M, Becker K, Walton-Moss B. Postpartum anxiety. J Nurse Practitioners. 2021;17(1):60–4. 10.1016/j.nurpra.2020.08.017. 10.1016/j.nurpra.2020.08.017

[43] Fox R, McMullen S, Newburn M. UK women’s experiences of breastfeeding and additional breastfeeding support: a qualitative study of Baby Café services. BMC Pregnancy Childbirth. 2015;15(1). 10.1186/s12884-015-0581-5. 10.1186/s12884-015-0581-5 PMC4494694 26148545

[44] Burns E, Schmied V, Sheehan A, Fenwick J. A meta-ethnographic synthesis of women’s experience of breastfeeding. Maternal Child Nutr. 2010;6(3):201–19. 10.1111/j.1740-8709.2009.00209.x. 10.1111/j.1740-8709.2009.00209.x PMC6860551 20929493

[45] Jackson L, De Pascalis L, Harrold J, Fallon V. Guilt, shame, and postpartum infant feeding outcomes: a systematic review. Maternal Child Nutr. 2021;17(3):e13141–n/a. 10.1111/mcn.13141 10.1111/mcn.13141 PMC8189225 33491303

[46] Beggs B, Koshy L, Neiterman E. Women’s perceptions and experiences of Breastfeeding: a scoping review of the literature. BMC Public Health. 2021;21(1):1–2169. 10.1186/s12889-021-12216-3. 34836514 10.1186/s12889-021-12216-3 PMC8626903

[47] Hoddinott P, Craig LCA, Britten J, McInnes RM. A serial qualitative interview study of infant feeding experiences: idealism meets realism. BMJ Open. 2012;2(2):000504–000504. 10.1136/bmjopen-2011-000504. 10.1136/bmjopen-2011-000504 PMC3307036 22422915

[48] Redshaw M, Henderson J. Safely delivered: a national survey of women’s experience of maternity care 2014. University of Oxford: National Perinatal Epidemiology Unit; 2015. https://www.npeu.ox.ac.uk/research/projects/119-national-maternity-survey-2014. [Accessed 10th June 2021].

[49] Karacam Z, Saglik M. Breastfeeding problems and interventions performed on problems: systematic review based on studies made in Turkey. Turk Pediatri Arsivi. 2018;53(3):134–48. 10.5152/TurkPediatriArs.2018.6350. 30459512 10.5152/TurkPediatriArs.2018.6350 PMC6239069

[50] Sambrook Smith M, Lawrence V, Sadler E, Easter A. Barriers to accessing mental health services for women with perinatal mental illness: systematic review and meta-synthesis of qualitative studies in the UK. BMJ Open. 2019;9:e024803. 10.1136/bmjopen-2018-024803. 30679296 10.1136/bmjopen-2018-024803 PMC6347898

[51] Mule V, Reilly NM, Schmied V, Kingston D, Austin MV. Why do some pregnant women not fully disclose at comprehensive psychosocial assessment with their midwife? Women Birth: J Australian Coll Midwives. 2022;35(1):80–6. 10.1016/j.wombi.2021.03.001. 10.1016/j.wombi.2021.03.001 33781709

[52] Wolgast E, Lindh-Åstrand L, Lilliecreutz C. Women’s perceptions of medication use during pregnancy and breastfeeding—A Swedish cross‐sectional questionnaire study. Acta Obstet Gynecol Scand. 2019;98(7):856–64. 10.1111/aogs.13570. 30739330 10.1111/aogs.13570

[53] University of Birmingham. Healthy Mum, Healthy Baby, Healthy Future The Case for UK Leadership in the Development of Safe, Effective and Accessible Medicines for Use in Pregnancy. University of Birmingham. 2022 https://www.birminghamhealthpartners.co.uk/wp-content/uploads/2022/05/Final-Healthy-Mum-Healthy-Baby-Healthy-Future-Report-AW_Accessible-PDF-REDUCED-FILE-SIZE.pdf [Accessed 11 June 2022].

[54] Borra C, Iacovou M, Sevilla A. New evidence on breastfeeding and postpartum depression: the importance of understanding women’s intentions. Matern Child Health J. 2015;19(4):897–907. 10.1007/s10995-014-1591-z. 25138629 10.1007/s10995-014-1591-z PMC4353856

[55] Kendall-Tackett K, Cong Z, Hale TW. The Effect of Feeding Method on Sleep Duration, maternal Well-being, and Postpartum Depression. Clin Lactation. 2011;2(2):22–6. 10.1891/215805311807011593. 10.1891/215805311807011593

[56] Manolova G, Waqas A, Chowdhary N, Salisbury TT, Dua T. Integrating perinatal mental healthcare into maternal and perinatal services in low and middle income countries. *BMJ* (Online). 2023;381, e073343–073343. 10.1136/bmj-2022-073343 10.1136/bmj-2022-073343 PMC10203867 37220917

[57] Sacks E, Finlayson K, Brizuela V, Crossland N, Ziegler D, Sauvé C, Langlois ÉV, Javadi D, Downe S, Bonet M. Factors that influence uptake of routine postnatal care: findings on women’s perspectives from a qualitative evidence synthesis. PLoS ONE. 2022;17(8):e0270264–0270264. 10.1371/journal.pone.0270264. 35960752 10.1371/journal.pone.0270264 PMC9374256

[58] Scarborough J, Norman A, Cooper L. The bidirectional relationship between breastfeeding and mental health. Br J Midwifery. 2022;30(10):554–62. 10.12968/bjom.2022.30.10.554. 10.12968/bjom.2022.30.10.554

[59] Frayne J, Ellies R, Nguyen T. Experiences of decision making about psychotropic medication during pregnancy and breastfeeding in women living with severe mental illness: a qualitative study. Arch Womens Ment Health. 2023;26:379–87. 10.1007/s00737-023-01325-0. https://www.doi-org.hallam.idm.oclc. 37171494 10.1007/s00737-023-01325-0 PMC10191939

